# BBB opening by low pulsed electric fields, depicted by delayed-contrast MRI, enables efficient delivery of therapeutic doxorubicin doses into mice brains

**DOI:** 10.1186/s12987-023-00468-7

**Published:** 2023-09-22

**Authors:** Itzik Cooper, David Last, Orly Ravid, Daniel Rand, Erez Matsree, Liora Omesi, Chen Shemesh, Meir Liberman, Leor Zach, Orit Furman, Dianne Daniels, Sigal Liraz-Zaltsman, Yael Mardor, Shirley Sharabi

**Affiliations:** 1 The Joseph Sagol Neuroscience Center, Sheba Medical Center, Ramat-Gan, 52621 Israel; 2 School of Medicine, Tel-Aviv University, Tel-Aviv, Israel; 3 School of Psychology, Reichman University, Herzliya, Israel; 4 The Advanced Technology Center, Sheba Medical Center, Ramat-Gan, 52621 Israel; 5 Oncology Institute, Tel Aviv Sourasky Medical Center, Tel-Aviv, Israel; 6 Department of Pharmacology, The Institute for Drug Research, The Hebrew University of Jerusalem, Jerusalem, Israel; 7 Institute for Health and Medical Professions, Department of Sports Therapy, Ono Academic College, Kiryat Ono, Israel

**Keywords:** Blood-brain barrier, Drug delivery, MRI, DCM, Pulsed electrical fields, Brain tumor

## Abstract

**Background:**

Pharmacological treatment of CNS diseases is limited due to the presence of the blood-brain barrier (BBB). Recent years showed significant advancement in the field of CNS drug delivery enablers, with technologies such as MR-guided focused ultrasound reaching clinical trials. This have inspired researchers in the field to invent novel brain barriers opening (BBo) technologies that are required to be simple, fast, safe and efficient. One such technology, recently developed by us, is BDF (Barrier Disrupting Fields), based on low pulsed electric fields (L-PEFs) for opening the BBB in a controlled, safe, reversible and non-invasive manner. Here, we conducted an in vivo study to show that BDF is a feasible technology for delivering Doxorubicin (Doxo) into mice brain. Means for depicting BBBo levels were developed and applied for monitoring the treatment and predicting response. Overall, the goals of the presented study were to demonstrate the feasibility for delivering therapeutic Doxo doses into naïve and tumor-bearing mice brains and applying delayed–contrast MRI (DCM) for monitoring the levels of BBBo.

**Methods:**

L-PEFs were applied using plate electrodes placed on the intact skull of naïve mice. L-PEFs/Sham mice were scanned immediately after the procedure by DCM (“MRI experiment”), or injected with Doxo and Trypan blue followed by delayed (4 h) perfusion and brain extraction (“Doxo experiment”). Doxo concentrations were measured in brain samples using confocal microscopy and compared to IC_50_ of Doxo in glioma cell lines in vitro. In order to map BBBo extent throughout the brain, pixel by pixel MR image analysis was performed using the DCM data. Finally, the efficacy of L-PEFs in combination with Doxo was tested in nude mice bearing intracranial human glioma tumors.

**Results:**

Significant amount of Doxo was found in cortical regions of all L-PEFs-treated mice brains (0.50 ± 0.06 µg Doxo/gr brain) while in Sham brains, Doxo concentrations were below or on the verge of detection limit (0.03 ± 0.02 µg Doxo/gr brain). This concentration was x97 higher than IC_50_ of Doxo calculated in gl261 mouse glioma cells and x8 higher than IC_50_ of Doxo calculated in U87 human glioma cells. DCM analysis revealed significant BBBo levels in the cortical regions of L-PEFs-treated mice; the average volume of BBBo in the L-PEFs-treated mice was x29 higher than in the Sham group. The calculated BBBo levels dropped exponentially as a function of BBBo threshold, similarly to the electric fields distribution in the brain. Finally, combining non-invasive L-PEFs with Doxo significantly decreased brain tumors growth rates in nude mice.

**Conclusions:**

Our results demonstrate significant BBBo levels induced by extra-cranial L-PEFs, enabling efficient delivery of therapeutic Doxo doses into the brain and reducing tumor growth. As BBBo was undetectable by standard contrast-enhanced MRI, DCM was applied to generate maps depicting the BBBo levels throughout the brain. These findings suggest that BDF is a promising technology for efficient drug delivery into the brain with important implications for future treatment of brain cancer and additional CNS diseases.

**Supplementary Information:**

The online version contains supplementary material available at 10.1186/s12987-023-00468-7.

## Introduction

Pharmacological treatment of brain tumors, either primary or secondary (metastatic), is limited due to the presence of the blood-brain barrier (BBB). The BBB helps to maintain brain homeostasis by limiting the trafficking of potential harmful compounds. In order for chemotherapy to be effective, it must cross the BBB in therapeutic doses. Although sometimes disrupted to some extent, the BBB is mainly intact in metastases and in localized parts of large tumors, thus hampering the ability of chemotherapy to effectively reach the cancerous cells [1].

Doxorubicin (Doxo), an anthracycline antibiotic, is among the most widely used anticancer agents. It inhibits the growth of many cancerous cells, including glioblastoma and breast cancer cell lines [2]. Despite significant antitumor effects in vitro, Doxo’s efficacy in brain tumors in vivo is limited by the BBB, as the presence of the efflux pump P-glycoprotein, expressed on brain endothelial cells, restricts Doxo entry into the brain [3, 4].

There are several methods to increase the concentration of Doxo in the brain or brain tumors. Using carriers such as liposomes or nanoparticles has shown some promise in animal studies [5], but the benefits for patients is still questionable. For example, in a phase 2 clinical trial from 2011, where patients with recurrent Glioblastome Multiforma (GBM) were treated with temozolomide and pegylated liposomal Doxo, the treatment group had no significant benefit over standard treatment [6]. In a recent clinical study [7] patients with recurrent GBM were treated with epidermal growth factor receptor (EGFR)-targeted immunoliposomes loaded with Doxo. Cerebrospinal fluid samples acquired from these patients showed no or negligible levels of Doxo, suggesting that Doxo did not cross the BBB. Doxo has shown some efficacy against brain tumors when administered directly into the tumor [8], however direct administration usually results in localized delivery, and may be less suitable for large, infiltrative tumors or for multiple metastases [9].

Opening the BBB in a controlled manner to increase drug concentration in the brain is a promising possibility. Localized minimal-invasive or invasive approaches include laser interstitial thermotherapy [10] and electroporation (EP, high intensity pulsed electric fields creating temporary pores in cells membranes which may increase the concentration of drugs in the cells or result in cell death) [11–13]. Non-invasive methods include hyperosmotic therapy [14], MRI-guided focused-ultrasound (MRgFUS) [15, 16] and low pulsed electric fields (L-PEFs) [17, 18]. We have previously demonstrated that L-PEFs, well below the threshold for EP, can transiently open the BBB [17, 18] in a dose dependent manner. We have further demonstrated that L-PEFs-induced BBB opening (BBBo) can be achieved using plate electrodes pressed against the intact skull of mice, suggesting this treatment has the potential to be non-invasive. BBBo was demonstrated by significant accumulation of Evans Blue dye in brain tissues and by delayed-contrast MRI (DCM), a method based on calculating the change in the signal of contrast-enhanced (Gadolinum-DPTA, Gd) T1-wighted MRI (T1-MRI) over time. Since the BBB mostly blocks Gd-based contrast agents from penetrating the brain, the DCM method is a sensitive, non-invasive and reliable method to depict BBBo that is characterized by slow Gd accumulation in the tissue.

We have previously demonstrated that combining minimally invasive EP with Cisplatin can delay tumors growth rates and improve survival in glioma baring rats [12]. The goals of the present study were: (1) To demonstrate that non-invasive L-PEFs, well below the threshold of EP, can be used to deliver therapeutic doses of Doxo, which otherwise does not pass the BBB, to the cortex of naïve mice; (2) To develop an MRI-based method for quantifying the extent of BBBo; (3) To demonstrate the potential of combining non-invasive L-PEFs with systemically administered Doxo to decrease tumors growth rates in mice.

## Methods

### Toxicity study in vitro

GL261 murine glioblastoma cells were obtained from the Leibniz Institute DSMZ-German Collection of Microorganisms and Cell Cultures (Braunschweig, Germany). U87 human glioma cells (U-87 MG) were purchased from ATCC. Cells were seeded on 96 well plates at 2,000 cells/well in growth medium composed of Dulbecco’s Modified Eagle Medium (DMEM, Biological Industries, Israel), supplemented with 10% fetal bovine serum, 1% L-glutamine, and 20 units/ml Penicillin-Streptomycin (ThermoFisher Scientific, Waltham, Massachusetts, USA), and maintained at 37 °C in a humidified incubator containing 5% CO_2_. The following day, Doxo (Teva pharmaceuticals, Israel) was added at increasing concentrations (n ≥ 6 wells/concentration) and confluence was automatically measured in real-time using Incucyte imaging system [19, 20] (Essen BioScience, Ann Arbor, MI, USA). Specifically, percentage of cell confluence was automatically calculated from four images taken every 3 h in each well, by the Incucyte analysis software. The resulting plot was fitted to a function using non-linear fit (GraphPrism 7.0.) and half maximal inhibitory concentration (IC_50_) was calculated.

### In vivo study

Animals were purchased from Envigo (Jerusalem, Israel) and the study was approved by the Sheba IACUC committee (1260/20 and 1321/21). Male Hsd mice, 9 weeks old, 25–30 gr, were used in this study. Prior to the experiment, the mice were kept at an animal facility with 12:12-h light-dark cycle. Food and water were provided ad libitum.

Prior to the experiments all animals were fully anesthetized (intramuscular injections of 250 µL of 1 mL/kg ketamine and 0.5 mL/kg xylazine) and remained under full anesthesia for the duration of the experiment. A venflon catheter was inserted into the tail vein prior to the procedure and was used for the administration of drugs and contrast agents.

**Fig. 1:**
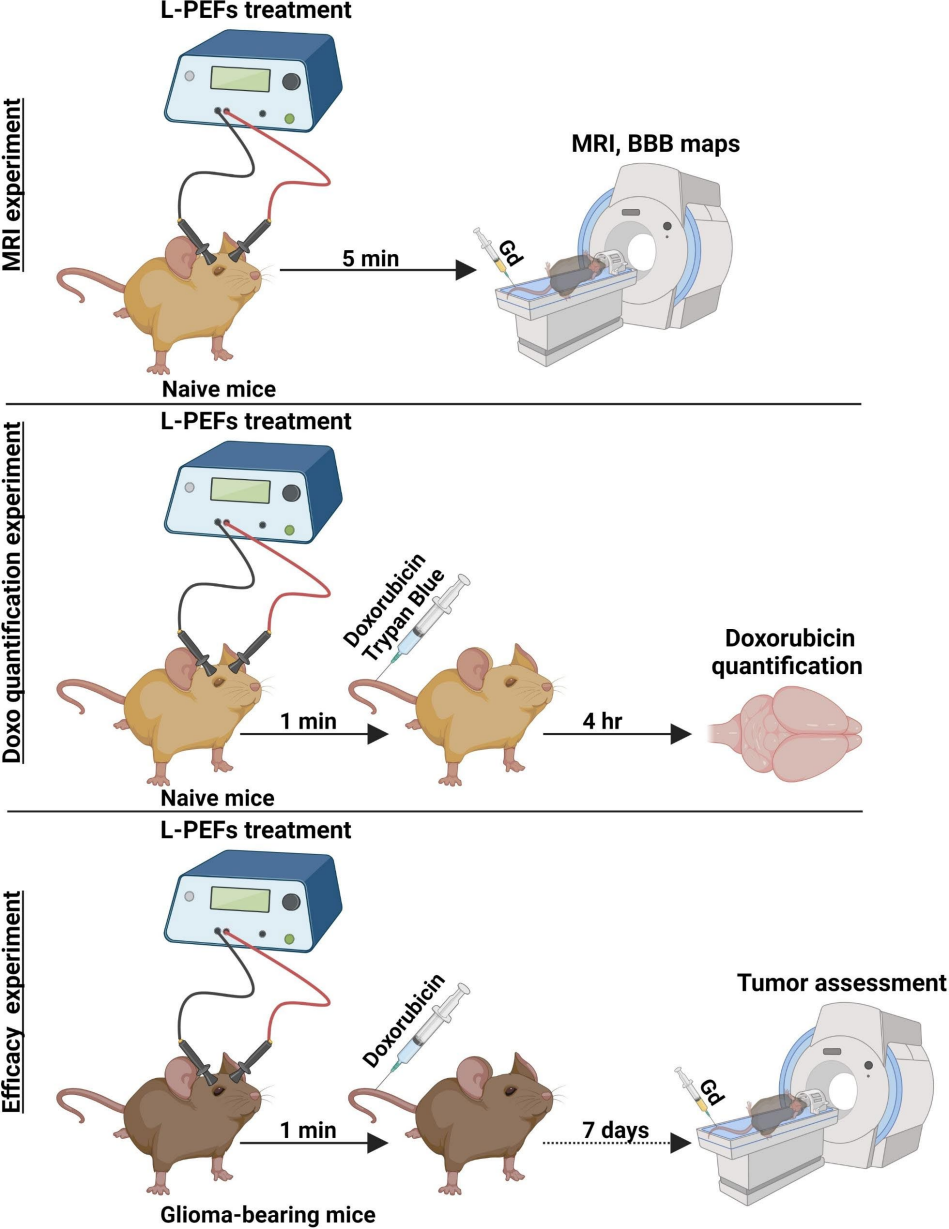
Experimental setup for the in vivo experiments (created with Biorender)

Three in vivo experiments were performed (Fig. 1): (1) “Doxo experiment”: 14 mice were injected post L-PEFs (n = 9)/Sham (n = 5) with Doxo and Trypan blue followed by intra-cardiac perfusion and brain extraction 4 h post L-PEFs treatment. (2) “MRI experiment”: 8 naïve mice were scanned immediately post L-PEFs/Sham (N = 4/group) procedure by DCM: repeated 3D T1-MRI were acquired up to 30 min post contrast injection. (3) “Efficacy experiment”: 10 intracranial tumor-baring nude mice were treated with L-PEFs and Doxo (n = 5) or Doxo only (n = 5). Tumor growth rates and response to treatment were studied using MRI.

L-PEFs parameters and procedure: Under full anesthesia (intramuscular injections of 250 µL of 1 mL/kg ketamine and 0.5 mL/kg xylazine), a midline scalp incision was made and the skull was exposed. A venflon catheter was inserted into the tail vein.

The L-PEFs procedure was applied using an electroporator (BTX 830; Harvard Apparatus, Holliston, MA). Two stainless steel 1.5 cm square plate electrodes (Caliper Electrode, Harvard Apparatus, Holliston, MA) were pressed against the sides of the intact skull after application of conductive gel (Abralyt HiCL, EASYCAP GmbH, Germany). The distance between the electrodes was kept at 1.2–1.3 cm.

Based on our previous optimization of L-PEFs treatment parameters [17], mice were treated with 100 pulses at 200 V. The pulse duration was 50 µs and the pulses were applied in sets of 25 pulses at a frequency of 4 Hz with 5 s intervals between sets. Sham procedures included anesthesia, skin incision, placing the electrodes and leaving the electrodes in place for 60 s.

#### Doxo experiment

Anesthetized mice were treated with L-PEFs. One minute post treatment, the mice were administered a mixture of 75 µl (6 mg/kg) Doxo Hydrochloride (Teva, Israel, MW:579.98 Da) [21] and 65 µl of Trypan blue (100 mg/kg) into the pre-installed venflon catheter. Trypan blue was administered to mark the disrupted areas for tissue extraction [21]. Prior to administration, the Trypan blue was dissolved in saline, boiled and filtered through a 0.2 μm membrane to avoid formation of microcrystals. When Doxo is administered for the treatment of brain tumors in rodents models, a single dose usually varies between 2 mg/kg -12 mg/kg [10, 22] and a weekly dose of 5 mg/kg for 5 weeks was found to be tolerable [23]. Here we chose to use 6 mg/kg to match this concentrations range [21].

Following 4 h of circulation, the mice were perfused with 80 ml of cold saline for 10 min (150 PSI). The brains were extracted and photos were captured. On ice, approximately 40 mg of tissue was harvested from cortical regions of the extracted brains; For the mice treated with L-PEFs, the harvested tissue samples were chosen both from regions clearly stained by the Trypan blue (stained/”blue”) and from regions where the dye intensity was lower (unstained/”white”). For the Sham mice, since no staining was visible (BBB was intact without L-PEFs treatment), approximately 40 mg of tissue was harvested from similar cortical regions. All the samples were weighed, immediately frozen in liquid nitrogen and kept at -80°C for further processing. In this experiment we used only 5 mice for the Sham group (in comparison to 9 for the L-PEFs group) since we anticipated very little Doxo penetration into the brain.

Doxo measurements in brain: Brain tissue samples were immersed in acidic ethanol (50% absolute ethanol in 0.3 N HCl) at 1:15 W/V ratio, homogenized using a bullet blender® with one ZroB20 2.0 mm bead/tube for 10 min and left for 24 h in 4°C. Samples were then centrifuged at 16,000 g for 25 min at 4°C. Two hundred µl were transferred from the supernatant into a clear 96 wells plate and fluorescence was measured and concentrations were calculated from a calibration curve; Fluorescent signal intensity was measured using confocal microscopy (Leica SP8) with a beam splitter function. The z plane was aligned to the bottom of the well and raised by 100 μm for every sample. A fixed set of parameters was used for all samples of a X20 lens and 5% 488 laser intensity.

#### MRI experiment

The aims of these experiments were: (1) Develop a non-invasive and clinically-relevant method to differentiate between contrast accumulation patterns, representing BBBo patterns, in order to predict extent of drug accumulation in the tissue via MRI in a region specific manner. (2) Demonstrate that spatial variability in Doxo concentration can also be assessed by MRI.

Approximately 2–5 min post L-PEFs application, the mice were scanned by DCM to evaluate BBBo. The MRI contrast agent (Gd-DOTA, 0.016 mmol/kg, Dotarem, Guerbert) was injected into the tail vein through the pre-installed venflon catheter inside the MRI, immediately prior to the first scan.

The mice were scanned with repeated 3D contrast-enhanced T1-MRI for at least 8 consecutive scans up to 30 min post contrast injection, as previously described [24].

MR image acquisition: The mice were scanned using a GE Optima scanner (1.5 T) with an 8-channel phased array wrist coil. T1-MRIs were acquired as following: 3D Cube T1-MRI: 10 cm FOV (phase FOV: 0.8), 244.14 kHz bandwidth, TE/TR = 21.4/602 ms, 0.8 mm slice thickness, 2562 matrix size, resulting in a voxel size of 0.39 × 0.39 × 0.4 mm^3^ after zerofill.

MR image analysis: In order to quantify the extent of BBBo, a pixel-by-pixel analysis was conducted: First, all the scans were co-registered to the first scan post contrast using rigid registration. Second, a region of interest (ROI) that included all the brain slices was plotted for each mouse. Then, the T1-MRI intensity of each brain pixel was normalized to the first time point intensity and was then plotted as a function of time post contrast injection (with a minimum of 7 time points post contrast captured during the first 30 min post contrast injection). The resulting plot was fitted to a 2-exponential function (Eq. 1), based on the 2-compartment exchange model of Tofts et al. [25–27].

1 $$x\left( 1 \right)*t*{e^{\frac{{ - t}}{{x\left( 2 \right)}}}} + x\left( 3 \right) + {e^{\frac{{ - t}}{{x\left( 4 \right)}}}} + x\left( 5 \right)\,\,x(3) * e - t/x(4)$$

Three parameters were extracted from the fit for each pixel:

1. Signal uptake (Dyn): computed as the difference between maximum intensity and the intensity of the 1st time point.
2. Time over threshold (TT): the duration for which the fit was above 5% signal intensity increase.
3. Area over the threshold (AOT): computed as the area under the curve and over the 5% signal intensity increase.

Only pixels with goodness of fit (r^2^) > 0.6, Dyn > 5%, and those included in a minimal cluster size of 1.9 cm^3^ (i.e. 30 pixels considering the image resolution) were considered to represent BBBo.

Next, for all the pixels representing BBBo in each mouse brain, histograms of the number of pixels were calculated for each parameter.

#### Efficacy experiment

Tumor inoculation: Anesthetized mice (intra muscular injections of 250 µL of 1 mL/kg ketamine and 0.5 mL/kg xylazine) were placed in a stereotactic frame. A midline scalp incision was made and the skull was exposed. A 1 mm burr hole was drilled 2 mm lateral and 1 mm anterior to the bregma. A 32-gauge needle connected to a syringe pump was inserted into the brain to a depth of 2 mm and was used to infuse 3 × 10^5^ U87 cells suspended in 3 µl PBS. The cells were infused at 0.5 µl/min for 6 min and the needle was kept in place for an additional minute and then extracted slowly from the brain. The burr hole was sealed with bone wax and biological glue was used to seal the incision. The mice were returned to the cages and metamizole was added to the drinking water for pain management [28].

Treatment and imaging: On day 5 after tumor inoculation the mice were anesthetized again and scanned by MRI in order to evaluate tumor size and location. The MRI contrast agent was injected intra peritoneal (Gd-DOTA, 0.125 mmol/kg, Dotarem, Guerbert) 20 min prior to MRI. Mice were then divided into two treatment groups (Doxo + L-PEFs and Doxo only) based on their tumor size, calculated from the enhancing volume on T1-MRI as described in the Image analysis section, so that the tumor size distributions were similar for the two groups. The tumor volumes calculated using the intraperitoneal contrast injections were only used for grouping purposes.

On day 6 from tumor inoculation (which will be referred to as the treatment day), mice were anesthetized as described above and were kept under full anesthesia for the duration of the procedure and MRI. A venflon was inserted into the tail vein for delivery of Doxo and Gd-DOTA. The skin incision was reopened and the skull was exposed in order to place the electrodes. Doxo (6 mg/kg, 150 µl diluted 1:1 in PBS) was injected into the tail vein and 1 min post injection L-PEFs treatment was applied (30 pulses at 300 V, 50 µs pulse duration at 4 Hz). Most of the tumors were seated in deeper locations than the cortex. We have previously shown that BBBo volume depends more on the voltage amplitude and less on the number of pulses and that increased penetration can be achieved with higher voltages [17]. For this reason, we increased the voltage amplitude in this experiment (300 V compared with 200 V used for the naïve mice). In order to compensate for the increased energy, the number of pulses was reduced to 30. Sham mice underwent similar procedure without activating the pulse generator. Immediately after L-PEFs the incision was sealed with biological glue and the mice were placed in the MRI. Gd-DOTA was injected immediately prior to the first scan and the mice were scanned with DCM as described above.

MR Image analysis: Tumor volumes were calculated from the first T1-MRI post contrast by plotting ROIs over the entire enhancing region in each slice. The volume of the tumors was then calculated by multiplying the number of pixels in the enhancing ROIs by the voxel volume. L-PEFs induced BBBo outside the tumor was calculated by plotting ROIs over the entire brain and subtracting from them the ROIs of the tumors. Next, BBBo volumes were calculated as described above for naïve mice and the volumes were compared between the two groups using student t’ test.

## Results

### Toxicity study in vitro

**Fig. 2:**
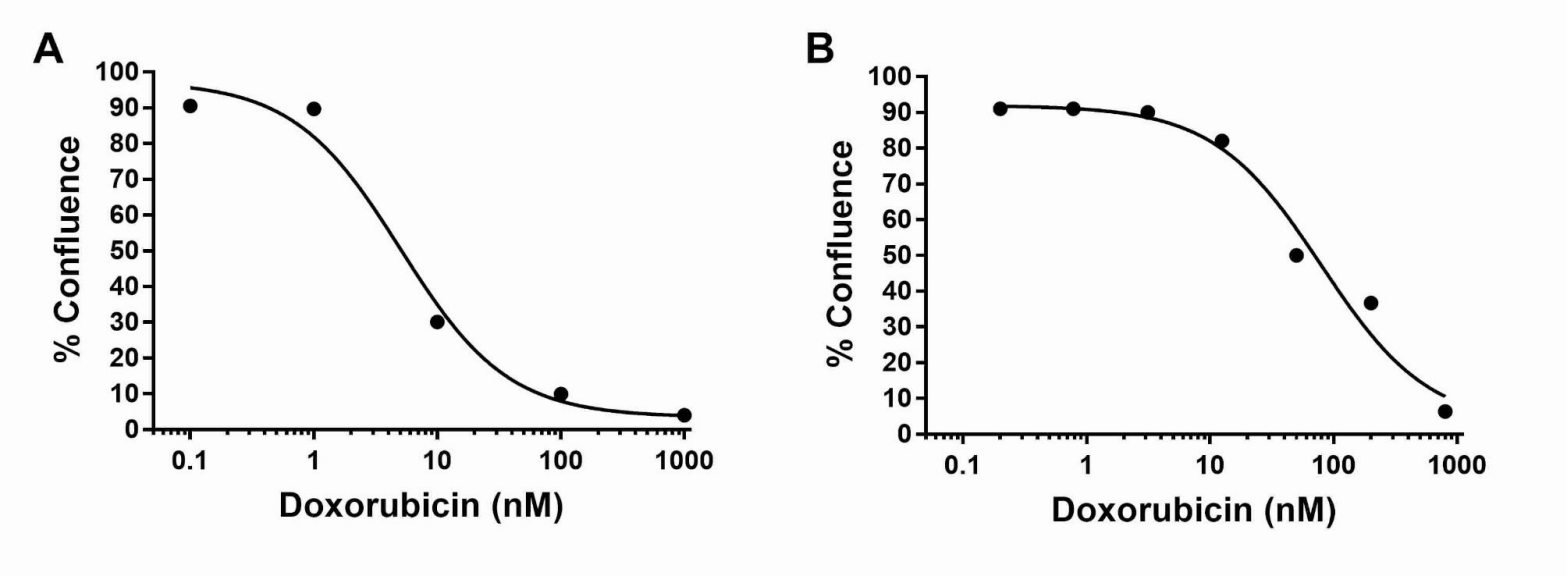
Doxo toxicity towards Gl261 and U87 glioma cells. Cells were seeded in a 96 wells plate and confluence was automatically measured in real-time using Incucyte. Four and single experiments for Gl261 **(A)** and U87 **(B)**, respectively, with n = 6 wells/concentration in each and 12 wells for control cells (vehicle) in each experiment. Shown are averaged percent confluence at day 5. Calculated IC_50_ are 7.1 ± 2.8 nM and 86.6 ± 10.0 nM (Average ± SEM) for Gl261 and U87, respectively (non-linear fit, GraphPad Prism7)

The effects of Doxo concentration on the confluence of mouse Gl261 and human U87 glioma cells were measured in real time in order to calculate the IC_50_ of the drug for the different glioma cell lines (Fig. 2). A dose-dependent graph was plotted for each cell line and IC_50_ was calculated. IC_50_ were found to be 7.1 ± 2.8 nM and 86.6 ± 10.0 nM for Gl261 and U87, respectively (non-linear fit, GraphPad Prism7). Micrographs and videos of the two cell lines treated with Doxo are presented in Supplementary Fig. 1 and Supplementary Videos 1a-c and 2a-c.

### In vivo studies

#### Doxo experiment

**Fig. 3:**
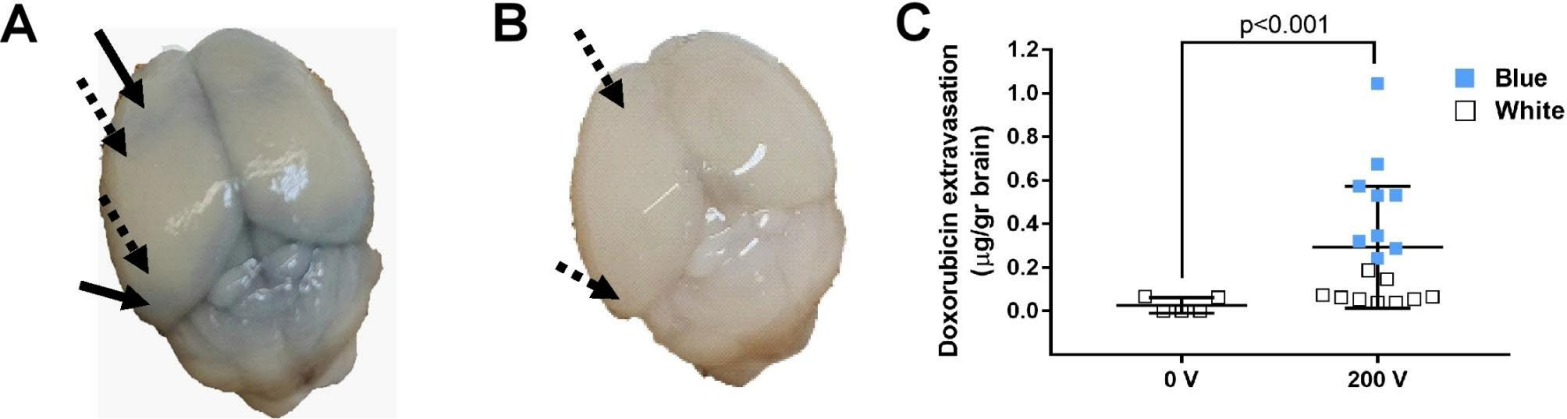
Doxo concentration in treated and untreated brains. **(A)** A brain of a mouse treated with L-PEFs (200 V). Arrows show where tissue was harvested (full arrow for stained (Blue) and dotted arrows for unstained tissues (White)). **(B)** A brain of a Sham mouse. Arrows show where tissue was harvested. **(C)** Average values of Doxo concentrations in treated (Blue/White) vs. untreated brain tissue. N = 5 for untreated mice, N = 9 for L-PEFs- treated mice

All 9 mice treated with L-PEFs showed spread areas of cortical BBBo as depicted by Trypan blue stains (Fig. 3A). Sham mice showed no signs of Trypan blue penetration (Fig. 3B). Doxo concentration in samples taken from mice treated with L-PEFs amounted to 0.3 ± 0.06 µg Doxo/gr tissue (Fig. 3C). These results include the Doxo concentrations of both stained and unstained samples. This concentration is translated to 690 nM considering brain tissue density (d_brain_=1.046). This is 97 fold higher than the IC_50_ of Doxo for Gl261 and 8 fold higher than the IC_50_ of Doxo for U87 (Fig. 2). In the Sham brains, Doxo concentrations were below or on the verge of detection limit (0.03 ± 0.02 µg Doxo/gr tissue). In 3/5 Sham samples Doxo was undetected. Thus, we found a significant difference between Sham samples and L-PEFs treated samples (student t test, p < 0.001, Fig. 3C). These results demonstrate the potential of L-PEFs for significantly increasing drug concentration in the brain.

In addition, a significant difference was found between Doxo concentrations in Trypan Blue stained and unstained samples collected from the treated mice brains (0.50 ± 0.06 µg Doxo/gr versus 0.09 ± 0.02 µg Doxo/gr tissue, respectively, student t test p < 3.2E-4). Although significantly smaller concentrations of Doxo were found in the unstained regions, Doxo was always detected. Significant variability was observed between the different samples harvested from the unstained areas compared to the Sham samples (range of 0.13 and 0.06 for the unstained L-PEFs-treated and Sham tissue samples, respectively, student t test p < 0.001, Fig. 3C).

#### MRI experiment

Standard T1-MRI, traditionally used to detect BBBo in brain lesions with significant BBBo (trauma, tumors etc.), showed no signs of contrast agent accumulation for all mice (Sham and L-PEFs treated). Nevertheless, DCM revealed significant contrast accumulation mainly in the cortical regions of the treated mice. These results are in accordance with our previous studies [17].

**Fig. 4:**
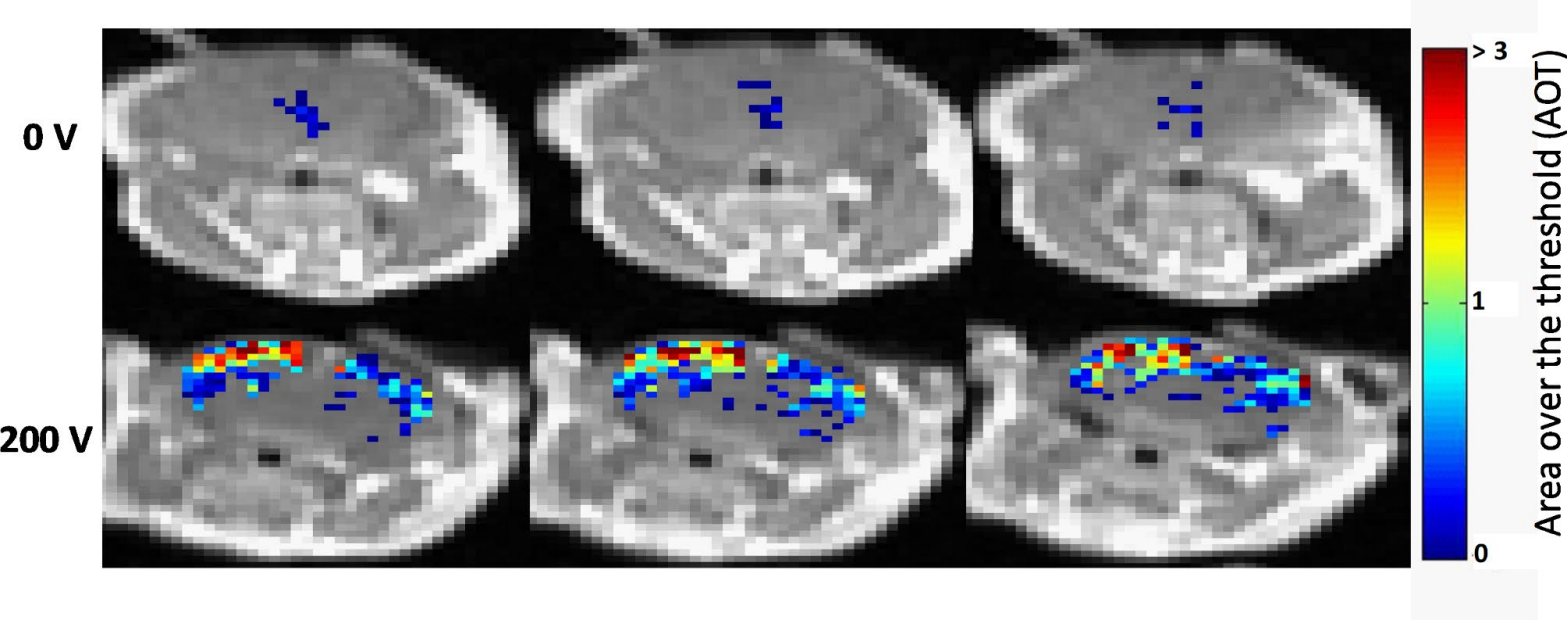
Representative T1-MR images showing BBBo-representing pixels. Three consecutive T1-MRI slices for 0 and 200 V superimposed with the area over threshold (AOT) parameter for all pixels presenting with BBBo. Representative images from N = 4 for both 0 and 200 V groups

As was previously described [24], a pixel by pixel MRI analysis was conducted resulting in significantly higher BBBo-representing pixels in the treated mice (1785 ± 351) versus the Sham mice (62.5 ± 30, student t test p < 0.016). In 2/4 Sham mice BBBo was undetected even by the sensitive DCM analysis. Additionally, for the treated mice, the majority of BBBo pixels were located in the cortical area, where the electric field induced by the L-PEFs treatment was highest, while in the Sham mice, the BBBo pattern was not localized to a specific brain region (Fig. 4).

**Fig. 5:**
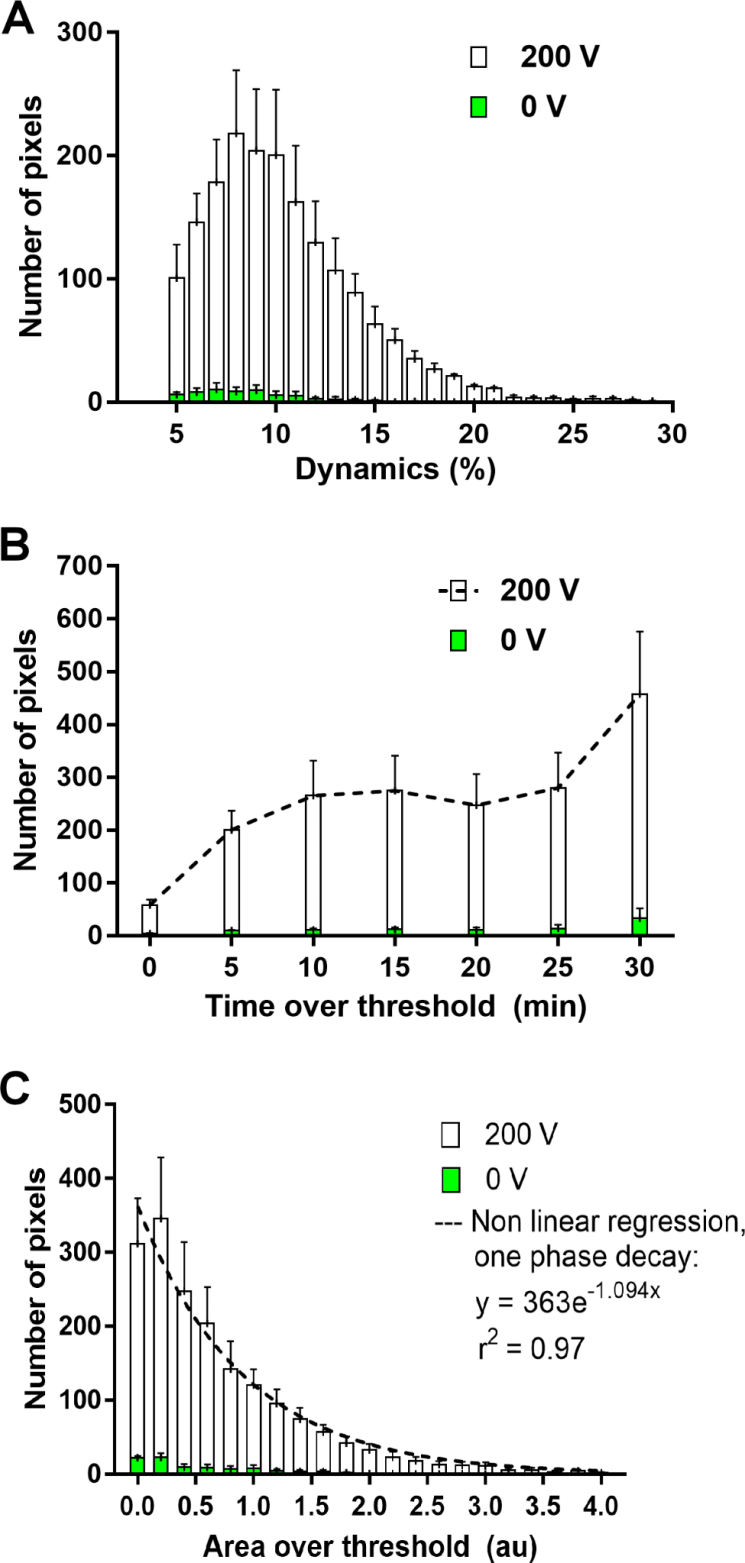
Histograms of the model parameters for all pixels representing BBBo at 200 V and 0 V. **(A)** Dynamic (Dyn): Maximal increase in signal intensity **(B)** Time over threshold (> 5% signal intensity, TT). **(C)** Area over the threshold (AOT). The results of this parameter were fitted to an exponential function. Shown are mean ± SEM from N = 4 for both 0 and 200 V groups

The level of the BBBo, which is directly linked to the number of drug/contrast molecules that pass from the blood vessels into the brain, depends mainly on the intensity (i.e. Dyn) and the duration of BBBo [29]. Increasing either the intensity or the duration of BBBo may result in an increase of the number of molecules passing into the brain. Here we calculated the duration of BBBo from the time of contrast injection, performed in the MRI a few min after the treatment (TT, Methods), and not the duration of entire BBBo window. Dyn and TT were extracted from the model for each pixel. In order to quantify the opening, histograms were plotted. Figure 5 A-B show the calculated histograms of Dyn and TT for all BBBo-representing pixels. The results show a bell shaped distribution for the Dyn, with over 50% of pixels showing Dyn of over 10%. The results show that the intensity of BBBo significantly varied between pixels. Similarly, variability can be observed for the TT, except that the histogram is skewed to the right, suggesting most of the pixels showed longer durations of BBBo.

As both parameters provide non-redundant indication of BBBo level, a parameter that incorporates both Dyn and TT is required. AOT includes the contributions of both parameters and also the slopes of the fit. Thus it may serve as a better predictor for BBBo level. Figure 5 C shows the histogram of average AOT for all mice that were treated with 100 pulses of 200 V. The results can be fitted to an exponential function (r^2^ = 0.97), suggesting that most pixels underwent low-medium BBBo and the number of pixels that underwent high BBBo dropped exponentially (50.4% of the pixels were below 0.6, 69.5% were below 1, 91.7% below 2 and 99% below 4). The exponential distribution of the AOT is similar to the exponential decay of the electric field in the brain from the brain surface to the center (data not shown).

#### Efficacy experiment

**Fig. 6:**
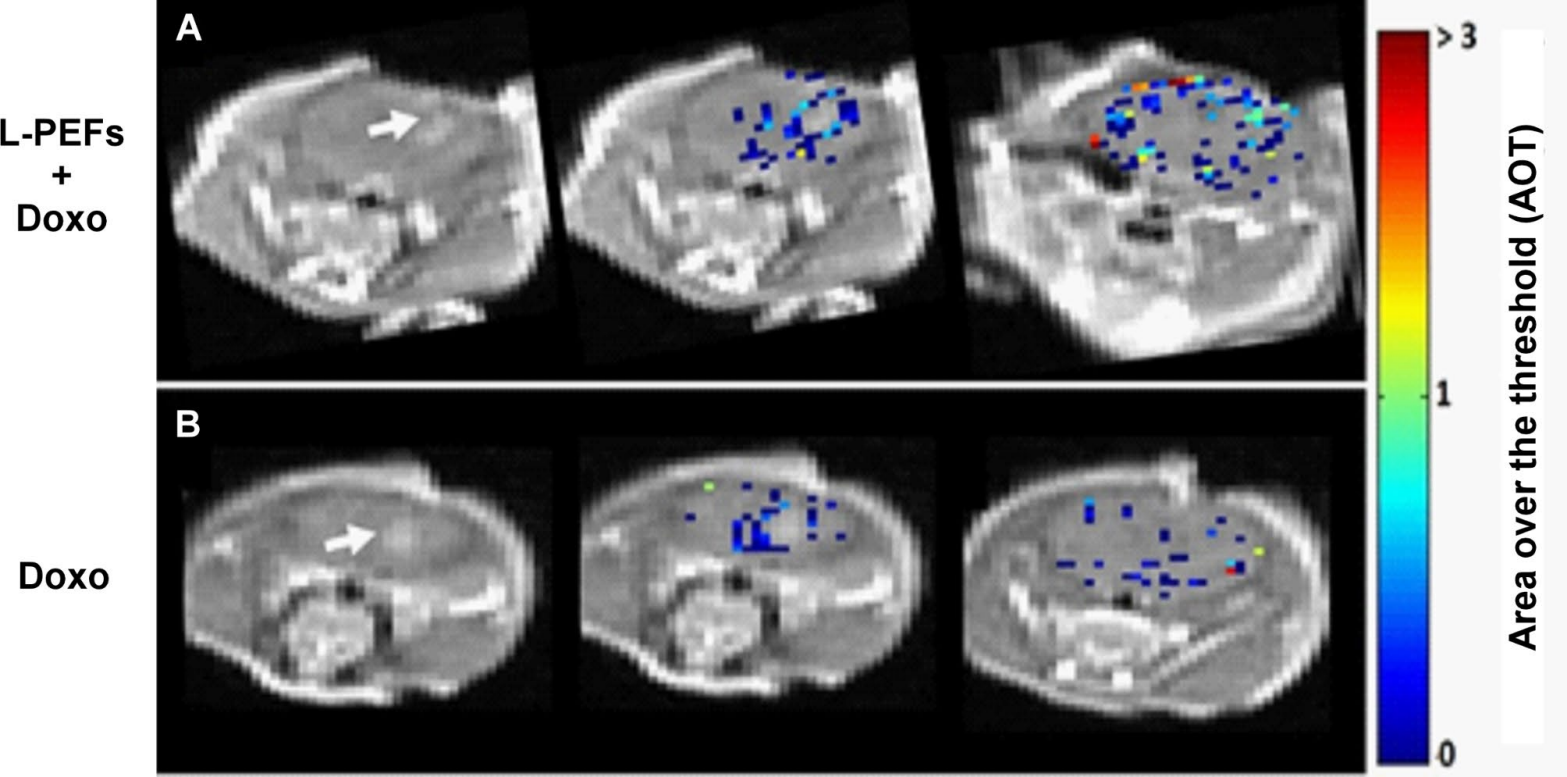
*Representative brain slices showing BBBo for the combined L-PEFs + Doxo group and the Doxo only group immediately post treatment*. A representative contrast-enhanced T1-MRI slice depicting the enhancing tumor (arrow) is shown on the left, and 2 slices showing pixels included in the area over the threshold (AOT), representing BBBo near the tumor and in further cortical areas, are shown on the right. **(A)** Combined L-PEFs + Doxo group (N = 5). **(B)** Doxo only group (N = 5)

Figure 6 shows BBBo in the two treatment groups of tumor-bearing mice (n = 5/group). One group was treated with L-PEFs (300 V, 30 pulses) + Doxo (combined treatment) and the second group was treated with Doxo only. Tumor volumes on the treatment day were 8.09 ± 3.47 mm^3^ and 5.76 ± 3.26 mm^3^ for the combined treatment and Doxo groups, respectively. No significant difference was found between the tumor volumes of the two groups before the treatment (student t’ test p < 0.65). The mice were rescanned on day 8 and growth rates were calculated for each tumor. A significant difference was found between the average tumors growth rates of the two groups after 8 days (1.18 ± 0.14 and 2.6 ± 0.44, for the combined treatment and Doxo groups, respectively, student t’ test p < 0.03), suggesting that the combined treatment of L-PEFs and Doxo significantly delayed tumor growth rates compared to the Doxo only group.

## Discussion

The BBB presents a major challenge for drug delivery into the brain, thus hampering the ability to efficiently treat brain diseases such as brain tumors. Doxo may serve as an efficient chemotherapy against brain tumors, however, it has low lipophilicity. In addition, Doxo is known as a substrate for the major efflux pump P-glycoprotein as well as other ATP-binding cassette transporters, including multidrug resistance proteins and breast cancer resistant protein (BCRP; ABCG2) [30]. These characteristics of Doxo result in nearly no penetration over the BBB, similarly to other chemotherapeutic agents. Combining chemotherapy with a BBBo technology in a controlled and safe manner has the potential to provide efficient drug delivery into the brain, thus significantly improving the prognosis of brain tumor patients [31].

L-PEFs is a method for transiently inducing BBBo by applying non-invasive extra-cranial electric fields in the order of several dozen V/cm [18, 24]. Our results show that applying L-PEFs using two plate electrodes pressed against the skull of a mouse (100 pulses at 200 V, 50 µs pulse duration at 4 Hz) can increase the penetration of intravenously administered Doxo into the cortex of naïve mice at therapeutic doses. Other electric fields based methods such as Electroporation (EP) utilize significantly higher electric fields (several hundreds to thousands V/cm) to induce BBBo, requiring surgical procedures which include craniotomy [32], thus presenting increased costs and complications for single and moreover repetitive treatments.

While L-PEFs affect the para-cellular route [18] and induce subtle and reversible BBBo, characterized by slow accumulation rates in the tissue [17], EP induces reversible BBBo by transient poration of the cells membranes [33–35]. Still, we have shown that BBBo induced by EP is accompanied by significant vasodilataion, causing fast washout of the drugs from the brain, which may result in low net drug concentrations in the tissue [11]. High frequency irreversible electroporation (HFIRE) utilizes high electric fields with shorter pulse durations than EP (0.5–10 µs) with BBBo lasting for 72 h [36]. The existence of cells membrane poration and the long duration of BBBo may lead to brain edema and inflammation [33, 37].

Magnetic resonance guided focused ultrasound (MRgFUS) is another non-invasive promising technology for inducing transient BBBo. During MRgFUS treatment, gas-filled microbubbles are injected intravenously and ultrasound pressure is applied to a target location in the brain, resulting in exertion of mechanical forces on the endothelial cells lining the brain vasculature [38]. Several effects including immediate opening of tight junctions were observed. BBBo lasts for up to 24 h and the treatment is in clinical trials for brain tumors, Alzheimer’s disease, and Amyotrophic Lateral Sclerosis (ALS) (NCT03739905, NCT03119961, NCT03671889). Despite showing promise, there are several secondary effects such as neuro-inflammation [39, 40], changes in brain transcriptome and proteome profiles, possible suppression of neuronal activity, changes in cerebral blood flow, and possible effects on clearance of metabolic waste products [38, 41]. We have previously demonstrated that L-PEFs induce no inflammation or edema [17].

We have previously demonstrated, using Evans Blue dye [17], that L-PEFs can increase the permeability of the BBB to protein bound molecules. Here, our goal was to demonstrate that L-PEFs can also be used to increase the permeability of the BBB to drugs that are also a target of the efflux pump P-glycoprotein and achieve therapeutic doses in the brain. Our results clearly demonstrate that this was achieved, as the average concentration in brain regions most affected by L-PEFs was 0.50 ± 0.06 µg Doxo/gr tissue. These Doxo levels are similar to the brain concentration achieved in rats using MRgFUS [42].

The distribution of Doxo in the cortex of L-PEFs treated mice was found to be non-uniform, with some areas showing significantly higher drug concentrations. This was seen both in DCM analysis and in brain tissue analysis. This non-uniformity can be attributed to the uneven distribution of electric fields in the tissue as the extent of opening depends on the strength of the electric fields [18]. We have previously shown that the electric fields created in the brain using this plate electrodes setup induces a non-uniform distribution [17].

The non-invasive DCM method also demonstrated non-uniform BBBo levels, supporting the Doxo experiment results. Further histogram analysis of the fit parameters extracted from the DCM method, specifically the AOT parameter, showed an exponential decay pattern. Similar decay pattern of the electric field as a function of distance from the electrode was described by Hjouj et al. [37]. This may strengthen the hypothesis that the difference in drug concentration between brain regions is correlated to the strength of the electric fields which these brain regions were exposed to. Regional differences in sensitivity to electrical fields in the brain, associated with different molecular patterns such as tight junction and adherence junction proteins distribution and organization, with additional difference in cytoskeleton sensitivity to electric fields may also result in a non-uniform BBBo [36, 43]. Future studies are needed in order to address and describe the correlation between the electric fields distribution, tissue specific molecular profiles and the level of BBBo.

The efficacy study combining L-PEFs with chemotherapy (Doxo) for the treatment of intracranial U87 tumors in a mouse model showed a significant reduction in tumor growth rates after a single treatment combining L-PEFs with Doxo compared to Doxo only. As expected, the volume of BBBo surrounding the tumor in the L-PEFs group was significantly higher than in the Doxo group immediately after the treatment, but no significant differences in BBBo were found on day 8, suggesting the effects of L-PEFs were transient. We have previously presented similar results in a study combining EP with Cisplatin for the treatment of intracranial CNS1 glioma tumors in rats [12]. Nevertheless, the EP experiment required drilling a burr hole in the skull and inserting an electrode through the brain tissue. Here we demonstrated that similar results can be achieved using L-PEFs while keeping the skull intact.

## Conclusions

Doxo is one of the most widely used chemotherapeutic drugs for the treatment of various solid tumors but is currently not used for the treatment of brain tumors [44]. Since Doxo was found to be a potent chemotherapy against glioma cells, it could have been extremely useful for brain tumors such as glioblastoma if it could reach the tumor cells. Our results suggest that the combination of this potent anti-cancer drug together with a safe, non-invasive and efficient BBBo technology such as L-PEFs, has the potential to improve clinical outcomes in patients with brain tumors. To reach clinical trials, additional in vivo studies should be performed, preferentially in larger animals. Alternatively, quantitative simulations for predicting barrier opening in the human brain, considering its unique structure and electric properties, can be performed to determine the optimal L-PEFs setup and parameters for treating humans. Our results further suggest that DCM, found to be a sensitive method allowing depiction of subtle changes in BBBo within specific brain regions, may be applied for monitoring BBBo levels thus predicting treatment response.

### Electronic supplementary material

Below is the link to the electronic supplementary material.

Supplementary Material 1: Figure 1. Micrographs of U87 and GL261 glioma cells. Microscopic pictures of the two cell lines treated with different concentrations of Doxo are shown at day 5. Bar = 400µm.

Supplementary Material 2: Video 1a. Growth of GL261 cells without treatment.

Supplementary Material 3: Video 1b. Growth of GL261 cells treated with 12.5nM Doxo.

Supplementary Material 4: Video 1c. Growth of GL261 cells treated with 50nM Doxo.

Supplementary Material 5: Video 2a. Growth of U87 cells without treatment.

Supplementary Material 6: Video 2b. Growth of U87 cells treated with 200nM Doxo.

Supplementary Material 7: Video 2c. Growth of U87 cells treated with 800nM Doxo.

## Data Availability

Data will be made available upon reasonable request.
