# Supplementary figures and images for: BBB opening by low pulsed electric fields, depicted by delayed-contrast MRI, enables efficient delivery of therapeutic doxorubicin doses into mice brains
Source: Fluids Barriers CNS. 2023 Sep 22;20:67. doi: 10.1186/s12987-023-00468-7 (PMC10515428; doi:10.1186/s12987-023-00468-7)

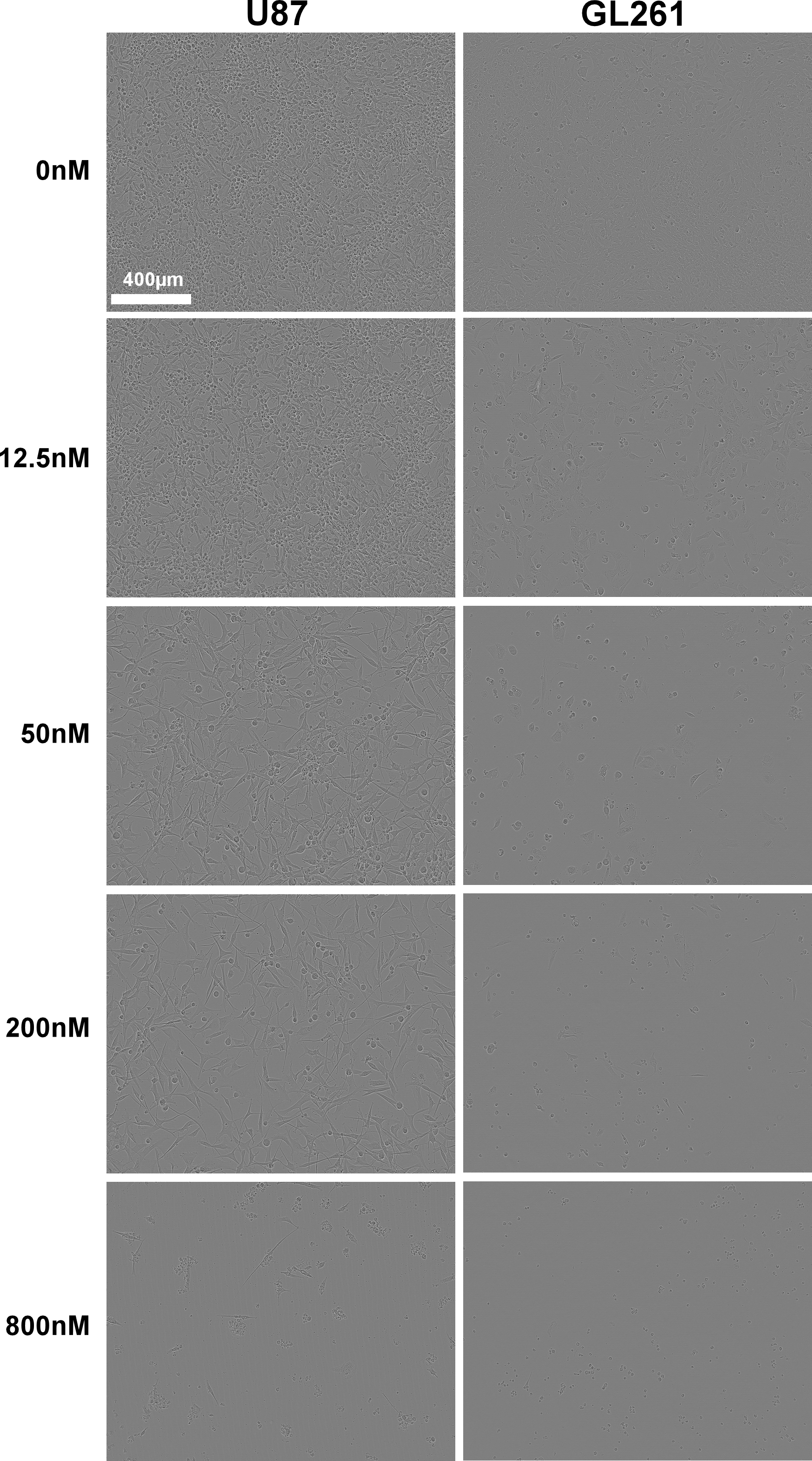

Supplement: Supplementary file 1 — Supplementary Material 1: Figure 1. Micrographs of U87 and GL261 glioma cells. Microscopic pictures of the two cell lines treated with different concentrations of Doxo are shown at day 5. Bar = 400µm. [file 12987_2023_468_MOESM1_ESM.jpg]
